# Heterogeneity matching and IDH prediction in adult-type diffuse gliomas: a DKI-based habitat analysis

**DOI:** 10.3389/fonc.2023.1202170

**Published:** 2023-11-28

**Authors:** Yanhao Liu, Peng Wang, Shaoyu Wang, Huapeng Zhang, Yang Song, Xu Yan, Yang Gao

**Affiliations:** ^1^ Department of Radiology, Affiliated Hospital of Inner Mongolia Medical University, Hohhot, China; ^2^ Magnetic Resonance Research Collaboration, Siemens Healthineers, Shanghai, China

**Keywords:** glioma, isocitrate dehydrogenase, diffusion magnetic resonance imaging, habitat, biomarkers

## Abstract

**Objective:**

To explain adult-type diffuse gliomas heterogeneity through diffusion kurtosis imaging-based habitat characteristics and develop and validate a comprehensive model for predicting isocitrate dehydrogenase (IDH) status.

**Materials and methods:**

In this prospective secondary analysis, 103 participants (mean age, 52 years; range, 21-77; 54 [52%] male) pathologically diagnosed with adult-type diffuse gliomas were enrolled between June 2018 and February 2022. The Otsu method was used to generate habitat maps with mean diffusivity (MD) and mean kurtosis (MK) for a total of 4 subhabitats containing 16 habitat features. Habitat heatmaps were created based on the Pearson correlation coefficient. The Habitat imAging aNd clinicraD INtegrated prEdiction SyStem (HANDINESS) was created by combining clinical features, conventional MRI morphological features, and habitat image features. ROC, calibration curve, and decision curve analyses were used to select the optimal model after 32 pipelines for model training and validation.

**Results:**

In the restricted diffusion and high-density subhabitat, MK was highly correlated with MD (R^2^ = 0.999), volume (0.608) and percentage of volume (0.663), and this region had the highest MK value (P<.001). The unrestricted diffusion and low-density subhabitat had the highest MD value (P<.001). When MK was less than the Otsu threshold, there was still a difference between restricted diffusion and low-density and unrestricted diffusion and low-density subhabitats (P<.01). The HANDINESS enabled more accurate prediction of the IDH status in the training (AUC=0.951 [0.902-0.987]) and internal validation cohorts (0.938 [0.881-0.949]). AUC values for single-modality models and independent factors ranged from 0.593 to 0.916. Calibration and decision curve analyses showed that the HANDINESS demonstrated a high level of clinical applicability and predictive consistency.

**Conclusion:**

Diffusion kurtosis imaging-based habitat analysis provides additional important information on microscopic tumor spatial heterogeneity. The HANDINESS has higher diagnostic performance and robustness than single-modality models.

## 1 Introduction

Adult-type diffuse glioma is a lethal brain tumor that demonstrates genetic, epigenetic, and environmental heterogeneity. Together, these heterogeneities constitute extreme phenotypic heterogeneity at the cellular level that provides multiple mechanisms for tumor cell hyperadaptation and treatment resistance (1, 2), resulting in poor patient prognosis. Furthermore, the 2021 WHO Classification of Tumors of the Central Nervous System emphasizes the important role of genetic heterogeneity in the evolutionary development of ltumors (3), particularly isocitrate dehydrogenase (IDH) heterogeneity. Differences in the IDH status can provide guidance in the selection of immunotherapy or targeted therapeutic approaches for oncology (4). Therefore, exploring inter- and intratumor heterogeneity associations as well as accurately predicting IDH status is crucial for developing personalized patient treatment regimens (2, 5).

In recent years, diffusion imaging techniques have become a topic of great interest in central nervous system disease applications, not only serving as a means of quantitative assessment of (6) spatial heterogeneity of tumors but also offering improvements to and complementing conventional MRI (cMRI). Exploring the heterogeneity of tumors remains a key focus and challenge in both scientific research and clinical practice. Diffusion kurtosis imaging (DKI) allows a more detailed assessment and response to the microstructural properties of living tissues (7) and has been implemented in various clinical applications (8, 9). DKI can effectively represent the non-Gaussian distribution of water molecule diffusion and quantify deviations from Gaussian diffusion. This characteristic is an advantage and aspect that is not possessed by cMRI. A summary of previous studies by Falk et al. (10) showed that the mean kurtosis (MK) and mean diffusivity (MD) in DKI have high diagnostic performance for tumor grading or gene prediction. The researchers suggested adding DKI to the routine imaging protocol for suspected glioma. However, in terms of describing the tumor spatial heterogeneity, the use of DKI alone remains inadequate.

Habitat imaging analysis is a method based on Darwinian evolutionary dynamics that combines information about tumor cells and their microenvironment to better reveal the spatial heterogeneity of tumors and essentially elucidate the laws controlling tumorigenesis and progression (11). In previous studies (12), glioma heterogeneity was analyzed to some extent by using habitat features, but the use of cMRI alone to create habitat maps may not be sufficient to explain the underlying mechanisms of tumor heterogeneity. We expect to extend the theoretical advantages of DKI by using habitat analysis, which is expected to overcome the possible shortcomings of previous studies (**Appendix S1**).

The purpose of this study was to explore the relationship between habitat maps and adult-type diffuse gliomas heterogeneity and to develop and validate a comprehensive predictive model of IDH status based on DKI habitat analysis techniques. This may help to explain the differences in the spatial heterogeneity of the glioma microenvironment and further enhance the accuracy of IDH status diagnosis in the clinic.

## 2 Materials and methods

We conducted a secondary analysis of the data obtained from a prospective study in accordance with the Declaration of Helsinki. The ethics committee of our hospital approved the study protocol, and all participants signed informed consent forms prior to enrollment in the cohort.

### 2.1 Participants and clinical data

**Figure 1:**
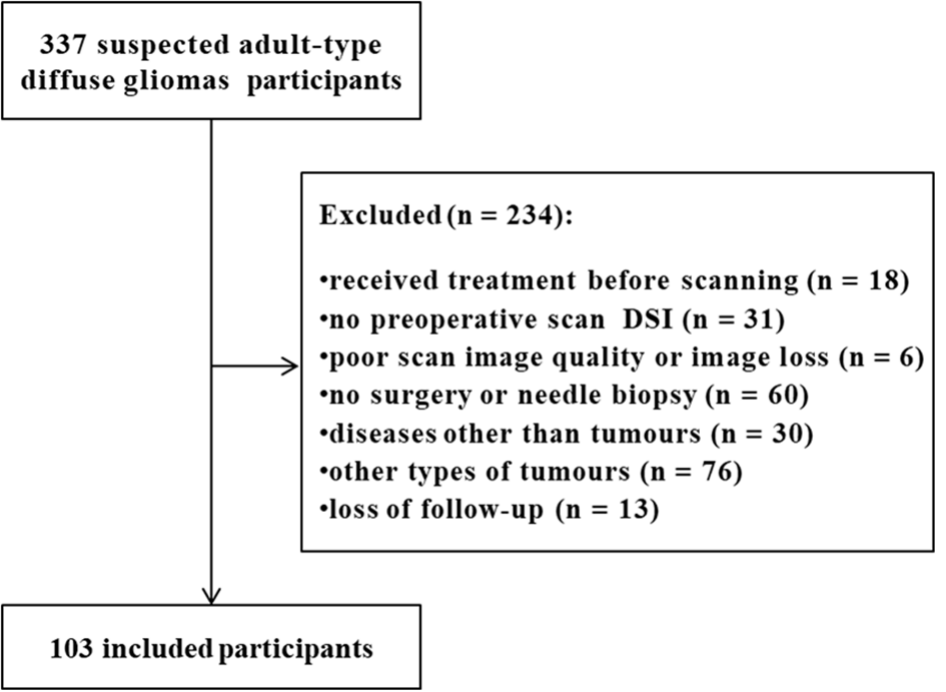
Flow chart of subject exclusion.

From June 2018 to February 2022, 337 subjects with suspected adult-type diffuse gliomas were registered in our hospital. All participants were suspected of adult-type diffuse gliomas due to clinical symptoms or previous imaging reports. We removed 234 patients who met the following exclusion criteria (1): treatment (including steroids, radiotherapy, chemotherapy, or concurrent radiotherapy and chemotherapy) before scanning or no preoperative diffusion spectrum imaging; (2) poor scan image quality or image loss; (3) no surgery or needle biopsy; and (4) a disease other than tumors or loss to follow-up. Finally, 103 eligible subjects (mean age, 52 years; range, 21-77; 54 [52%] male) were included in the cohort. Most participants received only general symptomatic care, such as a decrease in cranial pressure, between the MRI scan and surgery. **Figure 1** shows the participant inclusion process. According to the WHO Central nervous system Tumor Classification Criteria in 2021, astrocytoma subjects with IDH mutation and oligodendroglioma with IDH mutation and synchronous deletion of the short arm of chromosome 1 and long arm of chromosome 19 were classified as the IDH mutant group. Glioblastoma subjects with wild-type IDH were classified as the IDH wild-type group (**Supplementary Table S1**).

Fifty-five of sixty-seven subjects with adult-type diffuse gliomas from a previous study (13) were all included in the present study. The purpose of the previous study was to compare the accuracy and stability of 3 diffusion models, based separately on mean apparent propagator-MRI, DKI and diffusion tensor imaging, in predicting WHO grade and major genetic features in adult-type diffuse gliomas. The purpose of this study was to explore the potential mechanisms underlying the microscopic spatial heterogeneity of adult-type diffuse gliomas through macroscopic DKI habitat characteristics and to develop and validate a comprehensive model for predicting IDH status.

### 2.2 MRI scanning and preprocessing

All study participants underwent preoperative MRI using a 3T scanner (MAGNETOM Skyra; Siemens Healthcare, Erlangen, Germany) equipped with a 32-channel head/neck coil. To increase consistency in the radiographic characteristics, scans were carried out during the same time period (14). cMRI (T1WI, T2WI, FLAIR, CE-T1, DWI) and diffusion spectrum imaging were performed. Sequence details are shown in **Supplementary Table S2**.

Diffusion parameters were calculated using NeuDiLab, software developed in-house with Python based on the open-source tool Diffusion Imaging in Python (https://dipy.org) (15). The software is outfitted with FSL-based brain extraction, eddy-current and head-motion correction, and smoothing functions (16). ANTs were used for diffusion parametric mapping and cMRI alignment using default parameters in 3D-Slicer. The N4ITK MRI Bias correction module in 3D-Slicer was used to bias-correct the cMRI data (17).

### 2.3 Region of interest segmentation and habitat feature extraction

The regions of interest (ROI) were selected by two radiologists (ZY.H. and P.W., with 2 and 3 years of neuroimaging experience, respectively) using 3D-Slicer under the supervision of a more experienced doctor (Y.G., with 27 years of experience in neuroimaging). The three radiologists knew the diagnosis of the tumor but were blinded to the clinical and pathological details. The regions of interest were identified as areas affected by the tumor and were outlined on the B0 map using cMRI. Radiologically, the areas of solid tumor and peritumoral edema were typically reported as being encircled by abnormal/high signals on T2/T2-FLAIR. Areas near the edema that were thought to have been invaded, such as those with a slightly elevated signal or other unusual signal patterns discovered on T2WI, were typically also included in the region of interest.

**Figure 2:**
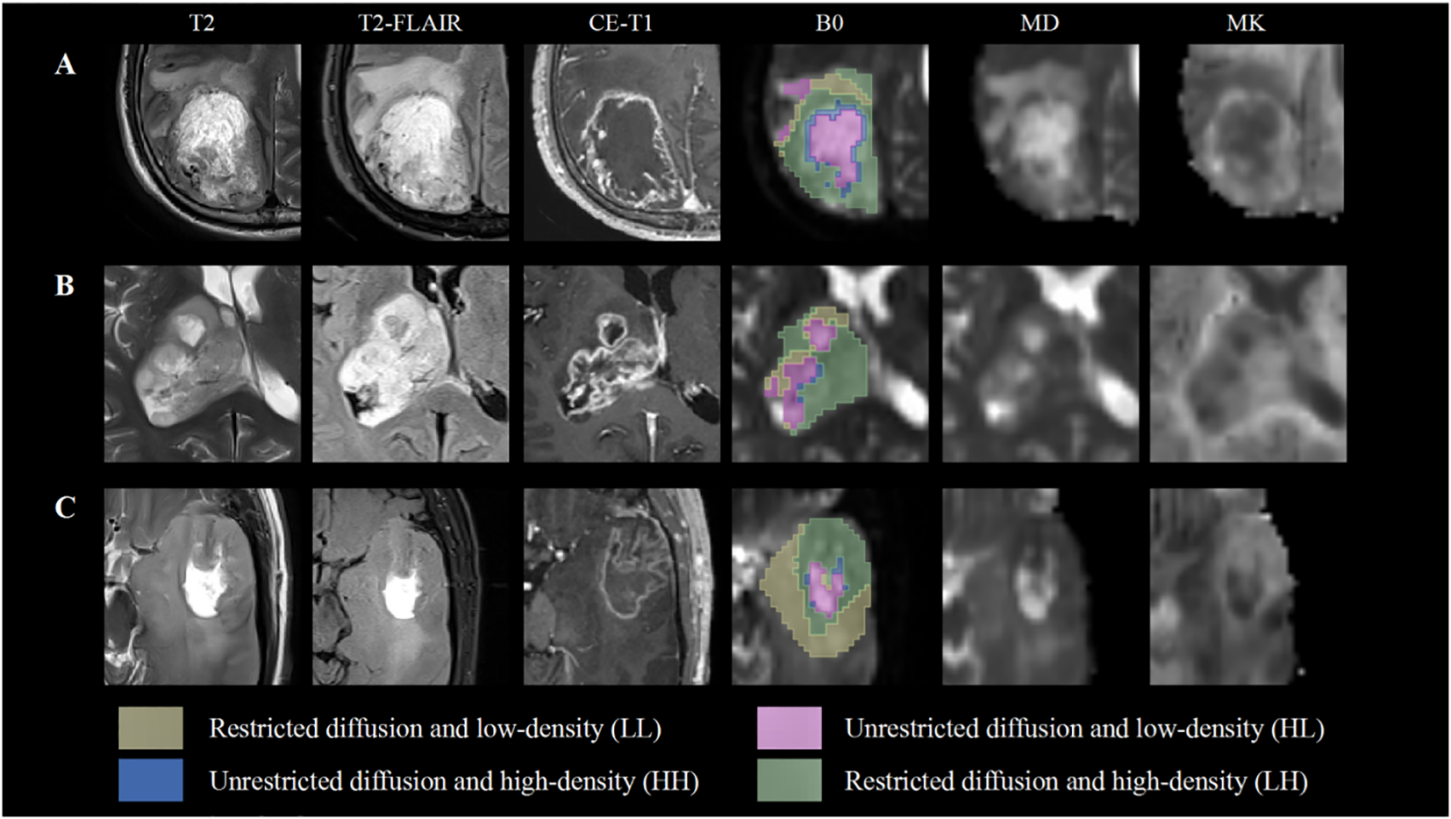
Subhabitats of three participants on different images. The B0 map shows four spatial habitats. The LL and HL habitats have a high level of overlap with peritumoral edema and cystic components compared with conventional MRI, mean diffusivity (MD) and mean kurtosis (MK) maps. **(A, B)** Show World Health Organization (WHO) grade IV glioblastoma with IDH wild type. **(C)** Indicates a WHO grade III oligodendroglioma with IDH mutation.

MD and MK maps were used as the main quantitative parameters (10, 18) for extracting habitat space features. The Otsu threshold method was applied to split the voxels into high- and low-intensity regions in the entire cohort for each map. The threshold value was determined iteratively to satisfy two conditions: 1) minimizing within-class variance and 2) maximizing between-class variance. The resulting subregions from each map were combined to obtain four final subregions: restricted diffusion and low-density (LL), restricted diffusion and high-density (LH), unrestricted diffusion and low-density (HL), and unrestricted diffusion and high-density (HH) subregions (**Supplementary Figure S1**). **Figure 2** demonstrates the high overlap between cMRI and habitat features, as well as the selection of ROIs. A total of 16 quantitative features, including MD and MK values, volume, and percentage of volume corresponding to the four subregions, were extracted. Intraclass correlation coefficients were used to assess the agreement between observers for the retrieved features. The habitats analysis was implemented by an in-house software nnFAE, which was developed based on python. The intraclass correlation coefficient values for the features were all 0.60 or above.

### 2.4 Extraction of morphological features

To evaluate the morphologic details of each participant, two radiologists independently examined all cMRI pictures (SH.X. and JL.H., with 11 and 12 years of neuroimaging expertise, respectively). The two radiologists were blinded to the clinical and pathological details but were aware of the tumor diagnosis. Additionally, judgments were divided into separate tasks that each included evaluation of a distinct feature by a radiologist who was unaware of the results of the other tasks. A senior physician (Q.W., with 15 years of neuroimaging expertise) was consulted when there was disagreement, and a final choice was then made. Imaging features were used to assess the solid tumor as well as any area of edema. Necrosis, cystic areas, calcification, the tumor enhancing pattern, location, and side as well as the clarity of solid tumor boundaries were all considered to be solid tumor components (19). The minimum length from the solid tumor to the adjacent white matter was evaluated in the peritumoral edema region. In **Appendix S2**, the classification standards used are displayed.

### 2.5 Correlation analysis and model construction

Pearson correlation analysis of the DKI-based habitat parameters was conducted with the aim of exploring potential associations between habitat diffusion parameters. Correlation coefficients between parameters were used to create heatmaps for further visualization of the data comparison. We classify correlations greater than 0.6 as high correlations. FeAture Explorer (FAE v0.5.2) (20) was used for model construction, and the optimal diagnostic model was selected. Thirty-two pipelines were used, including 2 dimensionality reduction methods (principal component analysis [**Supplementary Figure S2**] and Pearson correlation coefficients with a cutoff of 0.85), 4 feature selection methods (analysis of variance, recursive feature elimination, Kruskal–Wallis and Relief) and 4 modeling methods (logistic regression, least absolute shrinkage and selection operator, linear discriminant analysis, and support vector machine) (**Supplementary Table S3**). All subjects were included within the training group. Then, using the leave-one-out cross-validation, the diagnostic efficacy and stability with the internal validation cohort parameters were assessed.

### 2.6 Statistical analysis

All statistical analyses were performed using SPSS 24.0, R Version 4.1.2, and Python version 3.9.12(with Scikit-Learn as the primary). Quantitative data satisfying a normal distribution were compared using Student’s t test; otherwise, the Mann–Whitney U test was used. Precision-recall and receiver operating characteristic curve analyses were used to evaluate the performance of the model. Confidence interval (95% CI) calculations were performed using 1000 bootstrap intervals. The integrated discrimination improvement, net reclassification improvement, and DeLong test were used to assess the performance between the individual models. The calibration curve and Brier score were used to assess the agreement between predicted and actual probabilities across the models. Decision curve analysis was used to assess the net benefit. All statistical analyses were two-sided, with P<.05 indicating statistical significance. Details on the sample size and power calculations can be found in **Appendix S3**.

## 3 Results

### 3.1 Subjects in the study population

There were differences in age (P<.001), necrosis (<.001), hemorrhage (<.05), calcification (<.05), edema (<.05), tumor location category (<.001) and enhancement category (<.001) between the IDH mutation and IDH wild-type groups. There were no significant differences in sex, cysts, tumor borders, or tumor side (all P>.05). The characteristics of the participants are shown in **Table 1**.

**Table 1:** Participant characteristics.

Variable	IDH^mut^ (n=47)	IDH^wt^ (n=56)	P value
**Age (years)**	46.83±10.70	57.29±10.70	<.001
Sex*			.350
Male	27 (57.4%)	27 (48.2%)	
Female	20 (42.6%)	29 (51.8%)	
**Necrosis**			<.001
Present	26 (55.3%)	49 (70.0%)	
Absent	21 (44.7%)	7 (30.0%)	
**Hemorrhage**			.006
Present	28 (59.6%)	47 (83.9%)	
Absent	19 (40.4%)	9 (16.1%)	
**Calcification**			.009
Present	16 (34.0%)	7 (12.5%)	
Absent	31 (66.0%)	49 (87.5%)	
**Cyst or cysts**			.347
Present	40 (85.1%)	51 (91.1%)	
Absent	7 (14.9%)	5 (8.9%)	
**Edema (≤1.5 cm)**			.027
Yes	32 (68.1%)	26 (46.4%)	
No	15 (31.9%)	30 (53.6%)	
**Tumor borders**			.773
Sharp	23 (48.9%)	29 (51.8%)	
Blurry	24 (51.1%)	27 (48.2%)	
**Tumor location category**			<.001
Frontal or insula	37 (78.7%)	16 (28.6%)	
Other	3 (6.4%)	5 (8.9%)	
Basal nucleus or corpus callosum	7 (14.9%)	35 (62.5%)	
**Side**			.494
Left	22 (46.8%)	30 (53.6%)	
Right	25 (53.2%)	26 (46.4%)	
**Enhancement category**			<.001
Patchy enhancing	20 (42.6%)	5 (8.9%)	
Ringlike enhancing	16 (34.0%)	49 (87.5%)	
Nonenhancing	11 (23.4%)	2 (3.6%)	

Data are the mean ± standard deviation or n/N (%), where N is the total number of subjects with available data. P values were calculated with the chi-square test or Mann–Whitney U test.

^*^: Subject information was retrieved from the hospital registration system.

IDH^wt^, isocitrate dehydrogenase wild-type; IDH^mut^, isocitrate dehydrogenase mutant.

### 3.2 Distribution and correlation of habitat features

**Figure 3:**
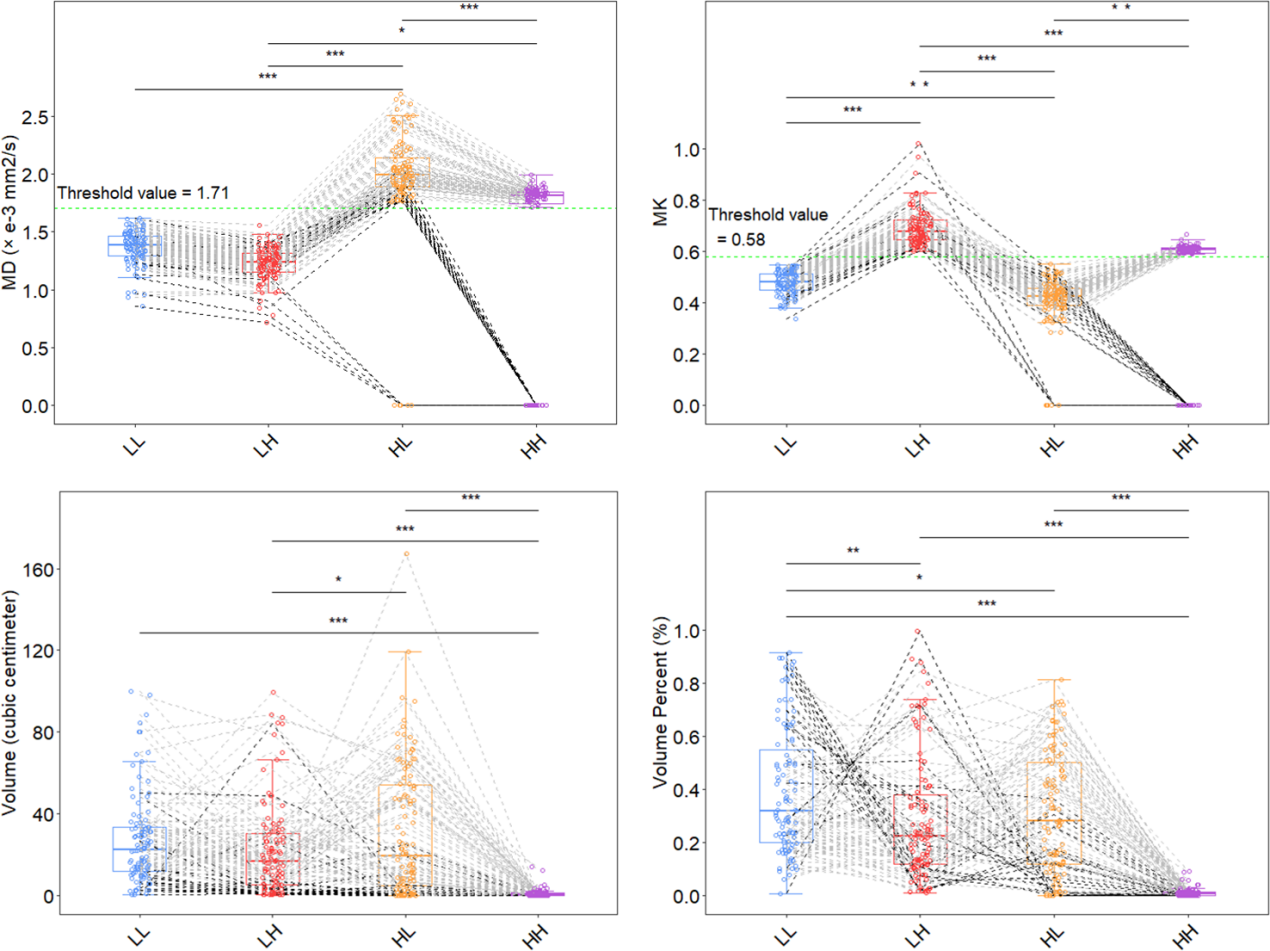
Distribution of features for each subhabitat. Two thresholds (MD 1.71 and MK 0.58; green dashed line) were used to divide the 4 habitat subregions. *P<.05, **P<.01, ***P<.001 using one-way ANOVA; multiple comparisons were adjusted for using the Bonferroni correction. The HL subhabitat was present in all IDH mutant subjects, and the HL subhabitat was absent in 5 IDH wild-type subjects. The HH subhabitat was absent in 15 IDH mutant subjects and 8 IDH wild-type subjects. The black dashed line represents the absence of the HH or HL habitat in the subjects, and the gray dashed line represents the other subjects. MK, mean kurtosis; MD, mean diffusivity; LL, restricted diffusion and low-density; LH, restricted diffusion and high-density; HL, unrestricted diffusion and low-density; HH, unrestricted diffusion and high-density; IDH, isocitrate dehydrogenase.

MD (P<.001), MK (<.001) and volume (<.05) were different between the LH and HL groups. MK (P<.001) and percentage of volume (<.001) were different between the LH and LL subregions. MD (P<.001), MK (<.01) and percentage of volume (<.05) were different between the LL and HL subregions. Among all adult-type diffuse gliomas participants, the parametric features of the HH habitats may be more concentrated. Subjects with an HH volume of 0 were included regardless of IDH status (**Figure 3**).

**Figure 4:**
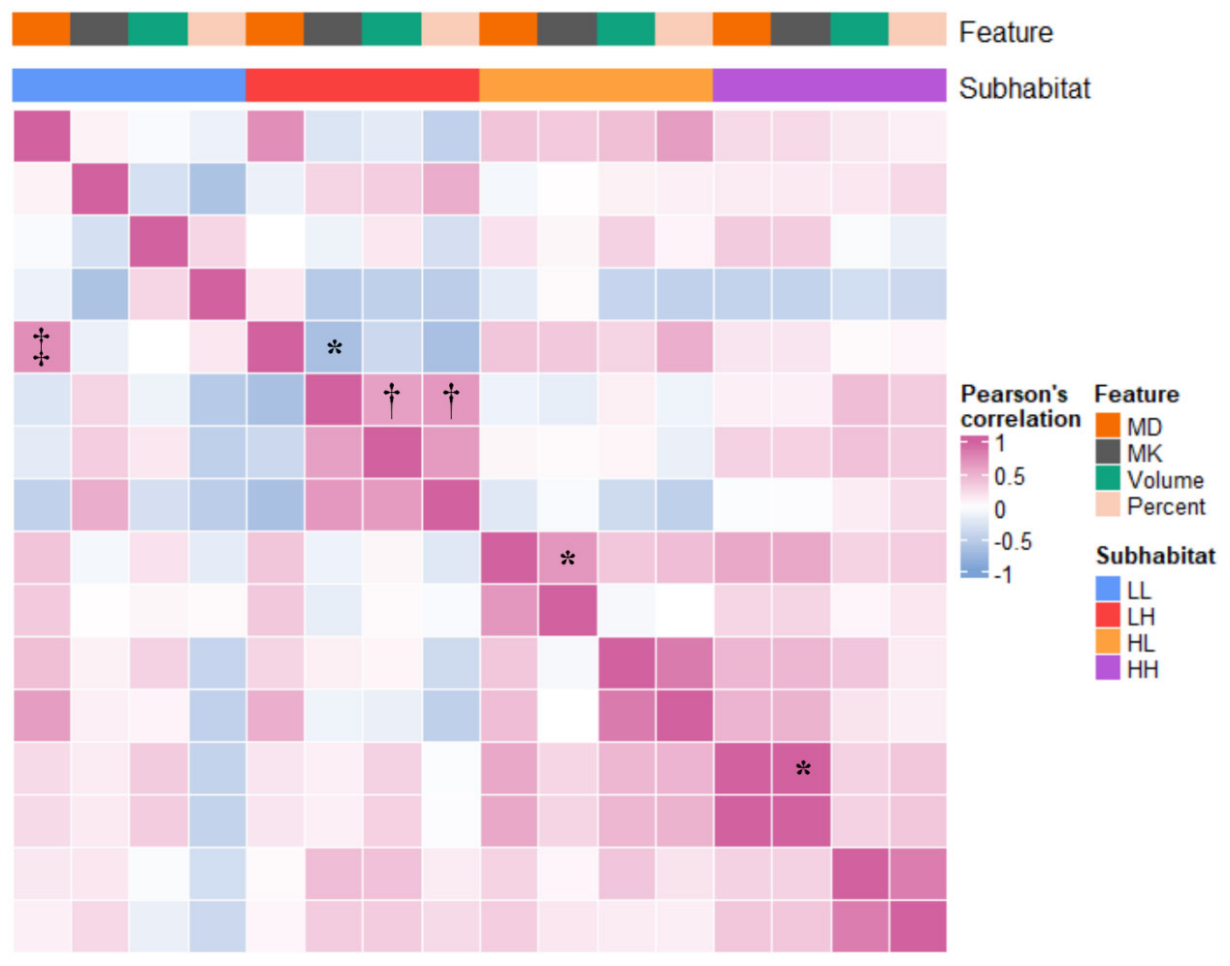
Correlation heatmap of diffusion parameters combined with habitat characteristics. Volume represents the volume of the subhabitat; percent represents the percentage of the total volume of the subhabitat. *Excluding the LL subhabitat, there was a high correlation between MK and MD in each habitat subregion (0.665-0.999). †Within the LH subhabitat, there was a high correlation between MD and volume and percentage of volume (0.608 and 0.663). ‡In the comparison between groups of habitat characteristics, only LL_MD and LH_MD were highly correlated (0.721). MK, mean kurtosis; MD, mean diffusivity; LL, restricted diffusion and low-density; LH, restricted diffusion and high-density; HL, unrestricted diffusion and low-density; HH, unrestricted diffusion and high-density.

High correlations were found between MK and MD in the LH (r=-0.666), HL (0.665) and HH regions (0.999) (all P<.001), and no correlations were found within the LL region (<.05). Only within the LH subhabitat was there a high correlation between MK and volume (r=0.608) and percentage of volume (0.663) simultaneously, and within the other independent habitats, there was a low (0.286-0.424) or no correlation (P>.05) or no simultaneous correlation between diffusion parameters and volume/percent. For the correlation analysis between habitat maps, a high correlation (r=0.721) was found only in the MD of the LL and LH subhabitats (**Figure 4**).

### 3.3 Establishment of three prediction models

Clinical and radiological score (ClinicRad), habitat analysis magnetic resonance imaging (H-MRI) and Habitat imAging aNd clinicraD INtegrated prEdiction SyStem (HANDINESS) models were constructed (**Supplementary Table S3**). The ClinicRad model was established by combining the clinical characteristics of the subjects and the imaging morphological features of the cMRI. The H-MRI model was built based on the habitat imaging analysis of DKI. Finally, we ensured that the habitat features had sufficient contributions to the model. The results of the H-MRI model were included in the construction of the HANDINESS model as an independent predictor along with the clinical features and imaging morphological features. The log odds ratios for the HANDINESS were calculated as follows: L= (0.03 × age) + (-0.65 × necrosis) + (-0.54 × hemorrhage) + (0.73 × tumor location category) + (1.65 × calcification) + (2.35 × H-MRI) + (0.48 × edema) - 6.14. The probability of IDH wild-type was then calculated for the HANDINESS model by using the equation 1/(1 + e-L); this value ranges from 0 to 1, with higher L values increasing the probability of IDH wild-type. The cutoff value provided by the HANDINESS was 0.38.

### 3.4 Performance and clinical value of the three models

**Figure 5:**
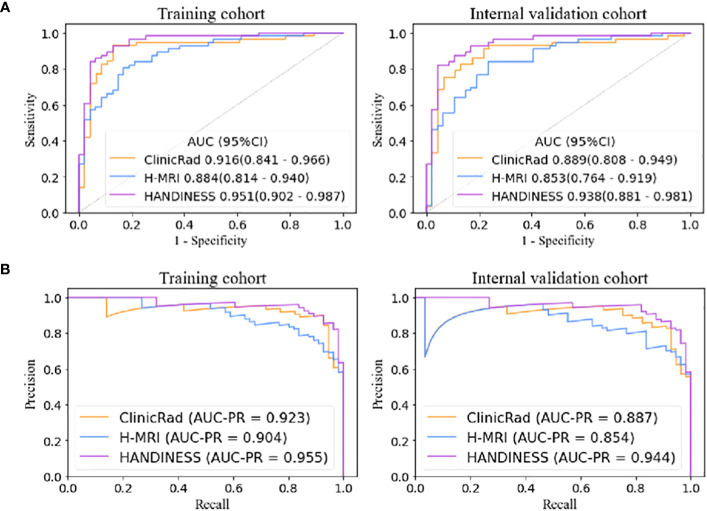
Receiver operating characteristic curves **(A)** and precision-recall curves **(B)** of the three models. The HANDINESS model had better diagnostic performance in the training and internal validation cohorts than the single ClinicRad model and the H-MRI model according to the integrated discrimination improvement (all *P*<.05). HANDINESS, Habitat imAging aNd clinicraD INtegrated prEdiction SyStem; H-MRI, habitat analysis magnetic resonance imaging; ClinicRad, clinical and radiological score.

HANDINESS predicted IDH status in glioma subjects more accurately both within the training and validation groups. The AUC of HANDINESS in the training group was 0.951 (0.902-0.987), accuracy was 0.893, sensitivity was 0.911, and specificity was 0.872. In the validation group the AUC was 0.938 (0.881-0.949), accuracy was 0.884, sensitivity was 0.821 and specificity was 0.957 (**Table 2**). The predictive performance of the HANDINESS model (0.951 and 0.938) was higher than that of the ClinicRad model (0.916 and 0.889) and the H-MRI model (0.884 and 0.853) (**Figure 5**). Regarding the areas under the precision-recall curves, the HANDINESS exhibited maximum values of 0.955 and 0.944 in the training and internal validation cohorts, respectively. In the AUC comparison diagram of the training cohort (**Supplementary Figure S3**), the AUCs for the single features of the ClinicRad model were 0.593-0.777, while that of the single factor of the H-MRI model ranged from 0.692 to 0.809.

**Table 2:** Prediction performance of the HANDINESS compared with the single-modality models.

	Training cohort	Internal validation cohort
HANDINESS		
AUC^*^	0.951 (0.902–0.987)	0.938 (0.881–0.949)
Sensitivity	0.911 (51/56)	0.821 (46/56)
Specificity	0.872 (41/47)	0.957 (45/47)
PPV	0.895 (51/57)	0.958 (46/48)
NPV	0.891 (41/46)	0.818 (45/55)
ACC	0.893 (92/103)	0.884 (91/103)
H-MRI		
AUC^*^	0.884 (0.814–0.940)	0.853 (0.764–0.919)
Sensitivity	0.786 (44/56)	0.839 (47/56)
Specificity	0.830 (39/47)	0.766 (36/47)
PPV	0.846 (44/52)	0.810 (47/58)
NPV	0.765 (39/51)	0.800 (36/45)
ACC	0.806 (83/103)	0.806 (83/103)
ClinicRad		
AUC^*^	0.916 (0.841–0.966)	0.889 (0.808–0.949)
Sensitivity	0.912 (52/57)	0.912 (52/57)
Specificity	0.870 (40/46)	0.783 (36/46)
PPV	0.897 (52/58)	0.839 (52/62)
NPV	0.889 (40/45)	0.878 (36/41)
ACC	0.893 (92/103)	0.854 (88/103)

Data in parentheses are the numerator/denominator of participants included for each parameter, unless otherwise indicated. Values correspond to the threshold according to the maximum Youden index.

*: Data are the mean (95% CI).

HANDINESS, Habitat imAging aNd clinicraD INtegrated prEdiction SyStem; H-MRI, habitat analysis magnetic resonance imaging; ClinicRad, clinical and radiological score.

The integrated discrimination improvement (all P<.05; **Supplementary Table S4**) and net reclassification improvement (all P<.05 except for the comparison between HANDINESS and ClinicRad in the training and validation cohorts; **Supplementary Table S5**) indicated that the HANDINESS model offered the highest diagnostic model efficacy. The excellent diagnostic performance of the HANDINESS was demonstrated by comparing the AUCs with the DeLong test (all P<.05 except for the comparison between the HANDINESS and ClinicRad models in the training cohort; **Supplementary Table S6**). There was no difference in diagnostic efficacy between ClinicRad and H-MRI.

We found differences in the P values between these three methods. The limitations of the DeLong test may be due to the small sample size. The selection of assessment nodes for risk stratification of data is not yet guided by highly reliable guidelines, which may be a limitation of the net reclassification improvement. Therefore, we believe that the P values from the integrated discrimination improvement are likely to be the most accurate.

Decision curve and calibration curve analyses suggested that the HANDINESS has better potential for clinical application and greater convergence in practical situations than the single-modality models (**Appendix S4**, **Supplementary Figure S4**).

## 4 Discussion

In this study, we described the potential relationship between DKI habitat features and microscopic anatomical regions of gliomas, with particular reference to the correlation with infiltrating tumor, cellular tumor. We then developed a comprehensive diagnostic model for the pretreatment prediction of IDH status in adult-type diffuse gliomas patients by combining quantitative clinical features, cMRI morphological features, and DKI habitat imaging features. The HANDINESS showed better prediction performance, both in the training cohort and the internal validation cohort (AUCs of 0.951 and 0.938, respectively) than the single prediction models.

The extraction of habitat features allowed the highly dense component of the cell (LH) to be effectively distinguished, independently of the LL component. We classified the LH subhabitat as a region highly similar to the cellular tumor (21), reflecting the nuclear heterogeneity of its malignant transformation by the high positive correlation between the microenvironmental volume and volume occupancy and the degree of cellular complexity. Differences due to histone methylation (22), epithelial growth factor receptor and mutation of the telomerase reverse transcriptase promoter (23) make the IDH wild-type tumor microenvironment more complex and cell dense, with more pronounced water molecule diffusion restriction, than that of IDH mutant tumors. This is reflected in our study by the high contribution of the LH habitat to the H-MRI model. A previous study showed (24) that MD and MK measurements in this region can help in prognostic assessment. Furthermore, the LH subhabitat is associated not only with IDH but also with the specific expression of TP53 and ATRX (21), which may provide additional targets of therapeutic relevance.

The LL subhabitat is an area of restricted diffusion and relatively low density that we speculate is mainly infiltrative tumor, which usually shows infiltrative edema with perineural satellite glial cell hyperplasia (21). This region has relatively limited water molecule diffusion because of various histopathological states, such as tumor cell value-added, vasogenic edema, micronecrosis, and extracellular matrix upregulation (25). In terms of macroscopic features, this zone is less complex than the LH subhabitat (cellular tumor zone), while the degree of water molecule restriction is closely related to that of the LH subhabitat (which can be interpreted as similar heterogeneity). The reason for the relatively low MK of the HL subhabitat is consistent with that of the LL subhabitat but differs in that the HL subhabitat has a more diffusion-unrestricted character. Considering the cMRI image features, possible explanations are that the LL and HL subhabitats have a large overlap with the peritumoral edema area of cMRI and that the HL subhabitat also contains part of the tumor cystic area. The HL habitat may contain simple vasogenic edema and cystic lesions. The hyperpermeable vessel wall causes an elevated MD with a decreased MK. A possible reason for not seeing areas of cystic lesions in anatomic subregions (21) may be that surgical resection usually does not preserve the complete tumor anatomy. Studies in the literature have suggested that the cyst fluid of malignant tumors may be a “trophic reservoir” with important determinants of growth of the surrounding tumor cells during tumor evolution (26) and is associated with tumor spatial heterogeneity. Therefore, the tumor cystic zone is still potentially valuable for research. In addition, it is difficult, if not impossible, to distinguish simple edema from infiltrative edema on cMRI, and DKI-based habitat analysis, which can differentiate infiltrative edema from other subregions, may be a good “weapon” for distinguishing peritumoral tumor cell from edema. This may help in the differential diagnosis of glioma from solitary brain metastases and lymphoma (27).

Some subjects did not have the HH subhabitat, regardless of their IDH status. This subhabitat may have similarities to the Gad-negative/low uptake subregion from a positron emission computed tomography study (28) and may reflect the hypoxic necrotic zone of the tumor, as in reality, this does not occur in all individual tumors. For the purpose of correctly reflecting the histological information provided by this habitat, tumor stereotactic biopsy is still required.

Although DKI parameters based on non-Gaussian distributions better characterize the high heterogeneity of gliomas than DWI and diffusion tensor imaging, the complex DKI model parameters alone (AUC 0.72-0.76) did not increase the accuracy in predicting IDH (29). We further visualized the macro- and microcorrelations (DKI-based habitat features with IDH) by means of habitat analysis to enhance the performance of the model. The interpretation of spatial heterogeneity in glioma using cMRI combined with habitat analysis remains somewhat lacking (30). The DKI-based habitat analysis showed a relatively high model performance (AUC 0.88). This is due to differences in cellular phenotype and genetic factors between wild-type IDH and mutant IDH (31), resulting in differences in regions of critical environmental selectivity and cellular adaptation. This link between gene status and habitat may produce a bidirectional channel between the molecular characterization of tumors and medical imaging as a way to improve the performance of the model. In addition, other diffusion indicators, such as fractional anisotropy and axial kurtosis, can reflect to some extent whether the integrity of brain fibers is disrupted (29), providing additional information on diffusion associated with glioma heterogeneity and suggesting an area of focus for future studies.

In general, IDH wild-type gliomas tend to be more malignant and more prone to hemorrhage and necrosis. Interestingly, the use of a single macroscopic factor, such as hemorrhage, enabled the prediction of poorer IDH performance in this study (0.63). This phenomenon was also observed in the study by Maynard et al. (19), where this feature did not improve the diagnostic efficacy of the model. The possible reason for this is that during the evolutionary progression of the tumor, hemorrhage due to vascular collapse and vessel wall lysis is associated with hypoxia or angiogenic factors (32), implying that hemorrhage is not a characteristic manifestation of tumor nuclear heterogeneity. Although these single factors reflect the complexity of the intratumoral and peritumoral spaces of glioma, the independent use of a single factor is not reliable. Notably, previous studies have also shown (9, 13) that the diagnostic performance when using a single parameter in diffusion tensor imaging or DKI in predicting IDH status is not satisfactory (AUC 0.74-0.86). HANDINESS, as one comprehensive model, which was constructed by fusing several features, showed the highest diagnostic value (AUC 0.95), reflecting the superior performance of the integrated model. A comprehensive model based on multiple parameters can accurately assess glioma IDH status, which will help guide the selection of clinical treatments and ultimately aid in prolonging patient survival cycles.

This study has some limitations. First, this study is based on a single-center sample collection. The sample size limits the valid analysis of the data, and the number of people in each group is not yet homogeneous, but the distribution is consistent with epidemiology. Second, the use of the Otsu method may not fully reflect the true pathophysiological cell density and spatial heterogeneity of the tumor; nevertheless, the method is not affected by image contrast, and the fast computation helps to balance the loss of efficiency from using segmented images. Third, the habitat analysis approach involving diffusion model development could only explore the heterogeneity of glioma in a single dimension, as there was a lack of information related to perfusion models or metabolic images. We have considered perfusion modeling for inclusion in subsequent studies (33). Finally, some new state-of-the-art diffusion models should be tried to be analyzed, even if short-term studies do not prove their practical superiority (34).

In conclusion, DKI-based habitat imaging analysis provides additional important information on the spatial heterogeneity of microscopic tumors. The HANDINESS multimodal prediction model had a diagnostic performance and robustness than the single-modality models. Exploration between macroscopic image features and microscopic tumor heterogeneity and the effective construction of comprehensive diagnostic models will help in future attempts to provide better personalized management and treatment planning for glioma patients in clinical practice.

## Nomenclature

### Resource Identification Initiative

NeuroImaging Tools and Resources Collaboratory (NITRC) (RRID:SCR_003430).

## Data availability statement

The original contributions presented in the study are included in the article/**Supplementary Material**. Further inquiries can be directed to the corresponding author.

## Ethics statement

The studies involving humans were approved by Ethics Committee of Affiliated Hospital of Inner Mongolia Medical University. The studies were conducted in accordance with the local legislation and institutional requirements. The participants provided their written informed consent to participate in this study. Written informed consent was obtained from the individual(s) for the publication of any potentially identifiable images or data included in this article.

## Author contributions

Guarantors of integrity of entire study, YG. Study concepts/study design or data acquisition or data analysis/interpretation, all authors. Manuscript drafting or manuscript revision for important intellectual content, all authors. Approval of final version of submitted manuscript, all authors. Agrees to ensure any questions related to the work are appropriately resolved, all authors. All authors contributed to the article and approved the submitted version.
