# Supplementary material for: Heterogeneity matching and IDH prediction in adult-type diffuse gliomas: a DKI-based habitat analysis
Source: Front Oncol. 2023 Nov 28;13:1202170. doi: 10.3389/fonc.2023.1202170 (PMC10713834; doi:10.3389/fonc.2023.1202170)
Supplement: Supplementary file 1 [file DataSheet_1.docx]

Supplementary Material

Heterogeneity Matching and IDH Prediction in Adult-type Diffuse Gliomas: A DKI-Based Habitat Analysis

Yanhao Liu^1,2,†^, Peng Wang^1,2,†^, Shaoyu Wang^3^, Huapeng Zhang^3^, Yang Song^3^, Xu Yan^3^, Yang Gao^1,*^

1 Department of Radiology, Affiliated Hospital of Inner Mongolia Medical University, Hohhot, China

2 Postgraduate School of Inner Mongolia Medical University, Hohhot, China

3 Magnetic Resonance Research Collaboration, Siemens Healthineers, Shanghai, China

†**First author**

The two authors contributed equally to this work.

*** Correspondence:**Yang Gao

1390903990@qq.com

# Supplementary Data

## Appendix S1. Research in context

### Evidence before this study

The intra- and intertumoral heterogeneity of adult-type diffuse gliomas greatly affects the treatment and prognosis of the tumor, and therefore the study of tumor heterogeneity is crucial. With no start time limit and an end date of November 1, 2023, we searched PubMed for publications that used spatial analysis based on medical imaging techniques to study the spatial heterogeneity of gliomas. We used the search terms ("Glioblastoma"[Mesh] OR "Glioma"[Mesh]) AND ("habitat Analysis" OR habitat* OR subregion* OR "Spatial Analysis"[Mesh]) without language restrictions. After excluding studies unrelated to habitat analysis methods, 28 original articles applying spatial analysis in the study of gliomas were retrieved, highlighting the potential relationship between spatial analysis and tumor microenvironmental heterogeneity. Twenty-six studies used magnetic resonance imaging as the core imaging modality, and the images used in 12 (46%) studies were obtained with conventional MRI (cMRI), mainly T1-enhanced and T2-FLAIR; 8 (31%) studies used diffusion imaging sequences, which helped in the interpretation of the tumor microenvironment. Five original diffusion imaging-based studies reported advances related to the use of spatial habitats to predict isocitrate dehydrogenase (IDH) mutation status in glioma, suggesting that the feature data extracted from habitat maps may be used as predictors. However, advanced diffusion models were not included in previous studies and may not reflect the more subtle pathophysiological changes of tumors. To our knowledge, no original study has prospectively explored the potential relationship between diffusion kurtosis imaging (DKI)-based habitat maps established according to the glioma microenvironment status and adult-type diffuse gliomas IDH mutation status.

### Added value of this study

This study applied the habitat analysis method based on diffusion kurtosis imaging to further visualize glioma microscopic spatial heterogeneity. Each subhabitat reflects the subtle microenvironmental changes that occur during the evolutionary progress of the tumor. High-risk habitats, such as restricted diffusion and high-density subhabitat, were highly correlated with glioma IDH status. Stereotactic tumor puncture using these high-risk habitats as image guidance may reduce the number of unnecessary and uninformative punctures. Additionally, subhabitats associated with peritumoral edema and tumor infiltration help to discriminate the nature of edema in adult-type diffuse gliomas. The Habitat imAging aNd clinicraD INtegrated prEdiction SyStem (HANDINESS) was constructed by combining quantitative clinical features, cMRI morphological features and DKI habitat features and was shown to predict the IDH mutation status in adult-type diffuse gliomas with high accuracy and robustness.

### Implications of all the available evidence

The potential of DKI-based habitat analysis methods to extract microscopic features of adult-type diffuse gliomas and predict IDH is introduced. Knowledge on the spatial heterogeneity of well-defined adult-type diffuse gliomas has important implications for future precise treatment. This may be of value in the development of individualized management plans for glioma patients.

## Appendix S2. Criteria for Conventional MRI Feature Extraction

The imaging features of cMRI were used to evaluate solid tumors and edema. Solid tumor components included necrosis, cystic regions, calcification, hemorrhage, the tumor enhancing pattern, position, and side as well as the clarity of the solid tumor borders. In the peritumoral edema area, the minimal distance was assessed.

1) Necrosis: Areas within the tumor body that were patchy or irregular in shape with signal intensity similar to cerebrospinal fluid; there were enhanced margins around areas of necrosis.

2) Cystic regions: Unlike necrosis, the signal was equivalent to the cerebrospinal fluid signal, and marginal enhancement was not significant or absent.

3) Calcification and hemorrhage: T1-weighted imaging, T2-weighted imaging, CT, and susceptibility weighted imaging (SWI) were used to determine the results (an additional CT or SWI scan was conducted in approximately 65% of the research individuals, which raises the level of uncertainty in the findings).

4) Tumor enhancement patterns: Contrast patterns were divided into three categories: patchy (degree of enhancement: slight), annular or central (degree of enhancement: significant), and no enhancement.

5) Location and side of the tumor: Based on where the middle of the tumor was located, a decision was made. The core of the lesion with the largest volume was examined when there were several lesions.

6) Boundary clarity: The tumor margin boundary was deemed to be fuzzy on T2-weighted imaging if more than 50% of the tumor margin area could not be properly delineated.

7) Edema extent: On T2-weighted imaging, the edema extent was determined in the largest aspect of the tumor entity. When the solid boundary of the tumor was clear (e.g., the tumor showed annular enhancement at the edge), the area of T2 hyperintensity around the tumor was considered edema. When the solid tumor and edema could not be distinguished, the T2 hyperintense areas that were closer in distance to the adjacent brain tissue and showed hyperintensity on apparent diffusion coefficient maps (areas of cytotoxic edema may be more characterized by unrestricted water molecules than solid tumors) were defined as edema. Finally, the minimum extent of the edema (perpendicular to the edge of the tumor) and whether it exceeded 1.5 cm was recorded. The minimum extent was chosen because it was likely to be more representative of tumor cell invasion, and the 1.5 cm threshold was derived from our clinical work experience.

## Appendix S3. Sample Size and Power Calculations

Approximately 70 participants will be consecutively enrolled in the research study. Regarding the AUC (H1), the study has ~85% power to detect an AUC of 0.80 for the HANDINESS model at an alpha value of 0.05 (two-sided) with 70 subjects. These assumptions served as a basis for the sample size computation: 1) the AUC in the null hypothesis was 0.60; and 2) the population with positive samples comprised 66% of the total. PASS 2021 software was used to calculate the sample size and power.

## Appendix S4. Clinical Value of the Three Models

Decision curve analysis (Supplementary Figure S4) showed that using the HANDINESS to predict the IDH status in glioma results in a higher net gain for subjects than the other two models, demonstrating the important role and benefit of the HANDINESS in this assessment. In the range of thresholds >0.1, the HANDINESS had more net benefits than ClinicRad and H-MRI models, with only a small overlap (0.3-0.4). With a minimal Brier score of 0.082, the HANDINESS demonstrated strong agreement between the predicted and observed outcomes in calibration curve analysis.

# Supplementary Figures and Tables

## Supplementary Figures

**
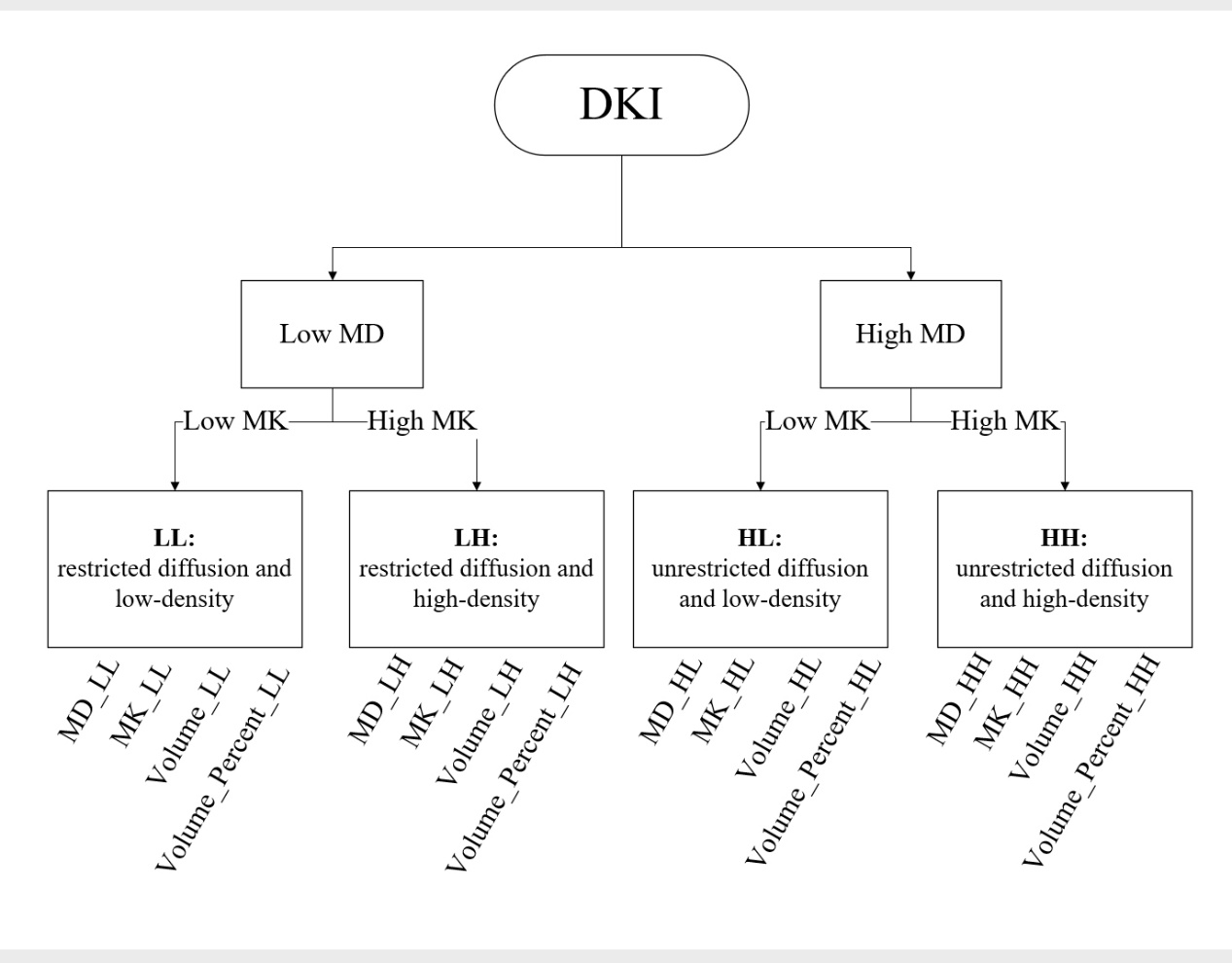
**

**Figure S1.** Habitat feature extraction flowchart

The Otsu threshold method split the voxels into high and low regions and then combined two subregions of each map to obtain the final four subregions.

Abbreviations: DKI = diffusion kurtosis imaging, MK = mean kurtosis, MD = mean diffusivity, LL= restricted diffusion and low-density, LH= restricted diffusion and high-density, HL= unrestricted diffusion and low-density, HH= unrestricted diffusion and high-density.


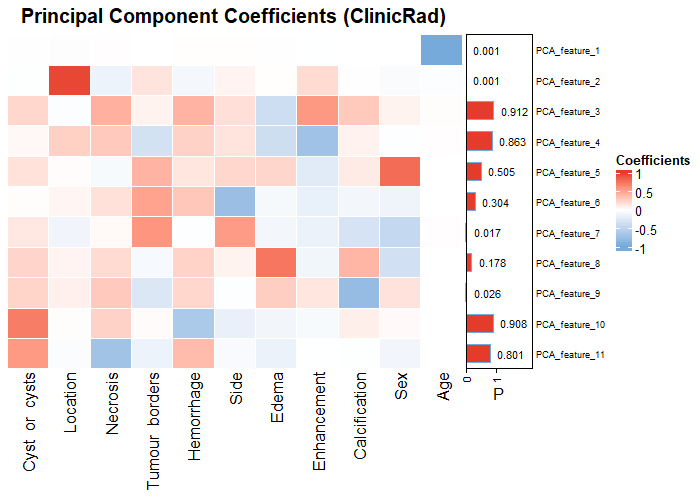


**Figure S2.** Principal component analysis of the ClinicRad model

Integration of clinical and morphological features of cMRI images into the ClinicRad model using principal component analysis.

Abbreviations: cMRI = conventional MRI, ClinicRad = clinical and radiological score.


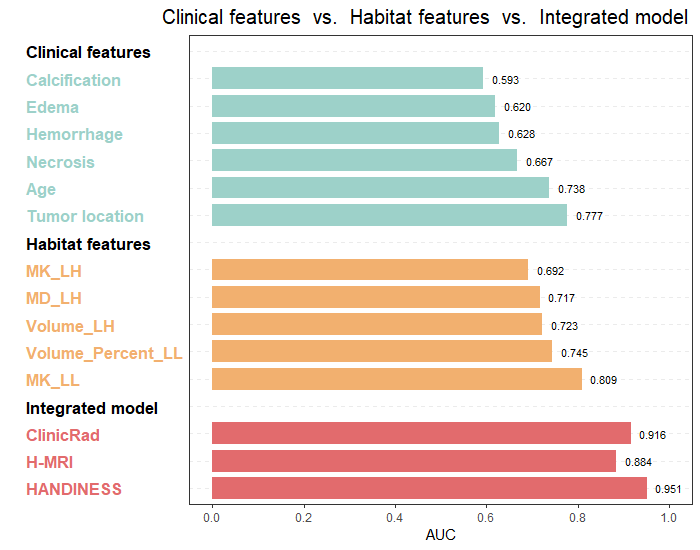


**Figure S3.** AUC comparison diagram of the training cohort

The comprehensive model (HANDINESS) achieved the highest diagnostic performance, with an AUC of 0.951, among all the models. The AUCs for single factors in the ClinRad model were 0.593-0.777. The AUCs for single factors in the H-MRI model were 0.692-0.809.

Abbreviations: AUC = area under the receiver operating characteristic curve, MK = mean kurtosis, MD = mean diffusivity, LL= restricted diffusion and low-density, LH= restricted diffusion and high-density, HL= unrestricted diffusion and low-density, HH= unrestricted diffusion and high-density, HANDINESS = Habitat imAging aNd clinicraD INtegrated prEdiction SyStem, H-MRI = habitat analysis magnetic resonance imaging, ClinicRad = clinical and radiological score.


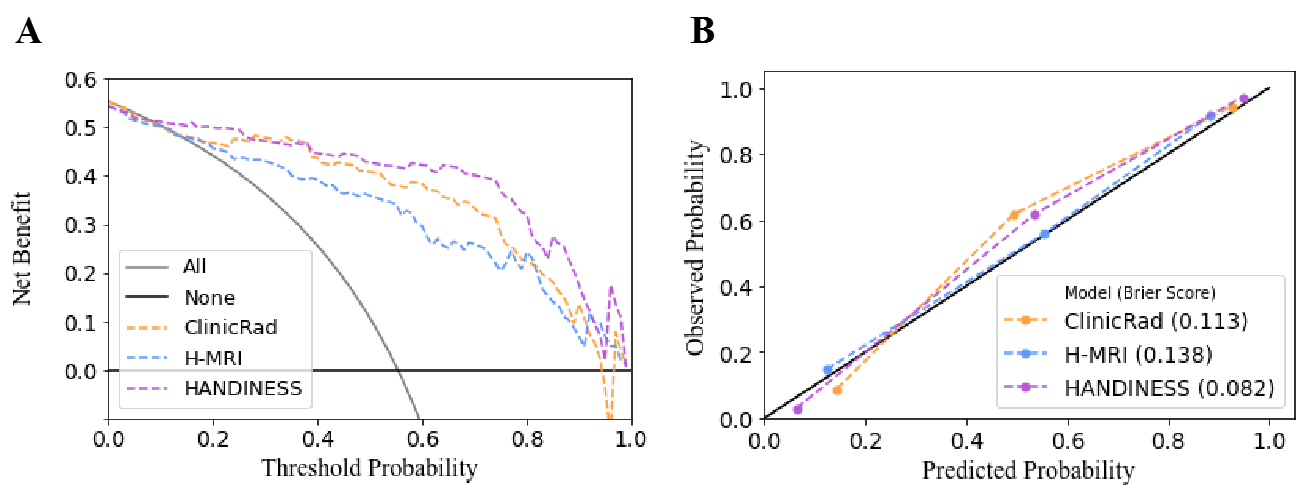


**Figure S4.** Decision curves (A) and calibration curves (B) of the three models

Higher values on the Y-axis (a) indicate a greater net benefit for patients. In the range of threshold values > 0.1, the HANDINESS yielded the highest net benefit, and only a small part of the ClinicRad model overlapped (0.3-0.4). Calibration curves (b) describe the calibration of each model in terms of agreement between the predicted results and the observed results. A higher degree of agreement between the dotted line and the solid line indicates a better prediction effect. The Brier score value ranges from 0 to 1, with smaller values representing better predictions.

Abbreviations: HANDINESS = Habitat imAging aNd clinicraD INtegrated prEdiction SyStem, H-MRI = habitat analysis magnetic resonance imaging, ClinicRad = clinical and radiological score.

## Supplementary Tables

**Table S1.** Adult-type diffuse gliomas classification

| **2021 WHO Integrated Diagnosis**  **(CNS WHO Grade)** | **IDH^mut^** | **IDH^wt^** |
| --- | --- | --- |
| Astrocytoma, IDH^mut^ (2) | 11 | - |
| Astrocytoma, IDH^mut^ (3) | 10 | - |
| Astrocytoma, IDH^mut^ (4) | 1 | - |
| Oligodendroglioma, IDH^mut^, and 1p/19q-codeleted (2) | 11 | - |
| Oligodendroglioma, IDH^mut^, and 1p/19q-codeleted (3) | 14 | - |
| Glioblastoma, IDH^wt^ (4) | - | 56 |

Note: Data are the mean ± SD or n/N (%), where N is the total number of study participants with available data. According to the 2021 WHO Classification of Tumors of the Central Nervous System, the classification of unfilled table spaces does not exist.

Abbreviations: CNS WHO Grade= Central Nervous System World Health Organization Grade, IDH^wt^ = isocitrate dehydrogenase wild-type, IDH^mut^ = isocitrate dehydrogenase mutant, 1p/19q = synchronous deletion of the short arm of chromosome 1 and long arm of chromosome 19.

**Table S2.** Anatomical and diffusion MRI parameters

|  | **T1** | **T2** | **T2-FLAIR** | **DWI** | **CE-T1** | **DSI** |
| --- | --- | --- | --- | --- | --- | --- |
| TA | 1 min 46 s | 1 min 45 s | 2 min 2 s | 1 min 4 s | 5 min 21 s | 15 min 40 s |
| TR (ms) | 1600 | 5500 | 6000 | 3530 | 2300 | 7000 |
| TE (ms) | 10 | 117 | 81 | 81, 128 | 2.32 | 107 |
| FOV (mm^2^) | 230*230 | 230*230 | 230*230 | 230*230 | 240*240 | 220*220 |
| Base resolution | 320 | 320 | 320 | 160 | 256 | 98 |
| Phase resolution | 75 | 100 | 70 | 100 | 256 | 98 |
| Voxel size (mm^3^) | 0.4*0.4*5.5 | 0.7*0.7*5.5 | 0.7*0.7*5.5 | 1.4*1.4*5.5 | 0.9*0.9*0.9 | 2.2*2.2*3.0 |
| Diffusion mode | - | - | - | 3-Scan Trace | - | q-Space |
| b-values (s/mm^2^) | - | - | - | 0, 1000 | - | 0-3000 |

The DKI model was constructed by DSI scanning. The construction of the model included 16 b-values (200, 350, 400, 550, 750, 950, 1150, 1500, 1700, 1850, 1900, 2050, 2250, 2450, 2650 and 3000 s/mm^2^) and 128 diffusion sampling.

Abbreviations: DSI = diffusion spectrum imaging.

**Table S3.** Selected features for model construction

| **Feature origin (N)^a^** | **Pipeline^c^ (dimension reduction/feature selector/classifier)** | **Feature name** | **Coefficient** |
| --- | --- | --- | --- |
| ClinicRad^b^ features  (N = 7) | PCA/KW/SVM | PCA_feature_1 | -0.09 |
|  |  | PCA_feature_2 | 1.22 |
|  |  | PCA_feature_5 | 0.23 |
|  |  | PCA_feature_6 | -0.36 |
|  |  | PCA_feature_7 | -1.41 |
|  |  | PCA_feature_8 | 1.26 |
|  |  | PCA_feature_9 | -0.80 |
| H-MRI^b^ features  (N = 5) | PCC/KW/LDA | MK_LH | -14.32 |
|  |  | MD_LH | -11.47 |
|  |  | Volume_LH | 0.01 |
|  |  | Volume_Percent_LL | -3.65 |
|  |  | MK_LL | 24.29 |
| HANDINESS^b^ features  (N = 7) | PCC/ANOVA/SVM | Rad score-H-MRI^d^ | 2.35 |
|  |  | Calcification | 1.65 |
|  |  | Edema | 0.48 |
|  |  | Hemorrhage | -0.54 |
|  |  | Necrosis | -0.65 |
|  |  | Age | 0.03 |
|  |  | Tumor location category | 0.73 |

Note: Thirty-two pipelines were used, including two dimensionality reduction methods (principal component analysis and Pearson correlation coefficients), four feature selection methods (analysis of variance, recursive feature elimination, Kruskal‒Wallis and relief) and four modeling methods (logistic regression, least absolute shrinkage and selection operator, linear discriminant analysis, and support vector machine). Finally, three optimal pipelines were selected to build the three models.

^a^: The total number of features in a distinct group.

^b^: Three different modeling approaches for identifying adult-type diffuse gliomas IDH status were included.

^c^: The processing of valid data features for modeling was called a pipeline.

^d^: The prediction results of the H-MRI model.

Abbreviations: PCA = principal component analysis, PCC = Pearson correlation coefficients, KW = Kruskal‒Wallis, ANOVA = analysis of variance, SVM = support vector machine, LDA = linear discriminant analysis, MK = mean kurtosis, MD = mean diffusivity, LL= restricted diffusion and low-density, LH = restricted diffusion and high-density, HANDINESS = Habitat imAging aNd clinicraD INtegrated prEdiction SyStem, H-MRI = habitat analysis magnetic resonance imaging, ClinicRad = clinical and radiological score.

**Table S4.** Integrated discrimination improvement in multiple cohorts

| **Model** | **Training cohort** | | **Internal validation cohort** | |
| --- | --- | --- | --- | --- |
|  | **IDI (95% CI)** | **P value** | **IDI (95% CI)** | **P value** |
| HANDINESS | / | / | / | / |
| H-MRI | 0.209 (0.098–0.319) | <.001^*^ | 0.175 (0.055–0.295) | .004^*^ |
| ClinicRad | 0.148 (0.062–0.234) | <.001^*^ | 0.119 (0.024–0.214) | .014^*^ |
| ClinicRad vs. H-MRI | 0.060(-0.063–0.185) | .140 | 0.056(-0.071–0.182) | .387 |

^*^: P <.05 indicated a difference between models.

Abbreviations: IDI = integrated discrimination improvement, HANDINESS = Habitat imAging aNd clinicraD INtegrated prEdiction SyStem, H-MRI = habitat analysis magnetic resonance imaging, ClinicRad = clinical and radiological score.

**Table S5.** Net reclassification improvement in multiple cohorts

| **Model** | **Training cohort** | | **Internal validation cohort** | |
| --- | --- | --- | --- | --- |
|  | **NRI (95% CI)** | **P value** | **NRI (95% CI)** | **P value** |
| HANDINESS | / | / | / | / |
| H-MRI | 0.256 (0.012–0.501) | .040^*^ | 0.252 (0.002–0.502) | .049^*^ |
| ClinicRad | 0.029 (-0.172–0.230) | .778 | -0.036 (-0.257–0.185) | .751 |
| ClinicRad vs. HMRI | 0.206 (-0.045–0.457) | .108 | 0.227 (-0.026–0.481) | .079 |

Note: Cutoff values for risk categories were defined as (0, 0.4, 0.7, 1).

^*^: P <.05 indicated a difference between models.

Abbreviations: NRI = net reclassification improvement, HANDINESS = Habitat imAging aNd clinicraD INtegrated prEdiction SyStem, H-MRI = habitat analysis magnetic resonance imaging, ClinicRad = clinical and radiological score.

**Table S6.** Delong test in multiple cohorts

| **Model** | **Training cohort** | | **Internal validation cohort** | |
| --- | --- | --- | --- | --- |
|  | **Difference between areas**  **(95% CI)** | **P value** | **Difference between areas**  **(95% CI)** | **P value** |
| HANDINESS | / | / | / | / |
| H-MRI | 0.068 (0.014–0.122) | .014* | 0.085 (0.023–0.148) | .008* |
| ClinicRad | 0.041 (-0.005–0.087) | .081 | 0.053 (0.001–0.105) | .048* |
| ClinicRad vs H-MRI | 0.027 (-0.058–0.112) | .539 | 0.032 (-0.065–0.130) | .516 |

^*^: P <.05 indicated a difference between models.

Abbreviations: NRI = net reclassification improvement, HANDINESS = Habitat imAging aNd clinicraD INtegrated prEdiction SyStem, H-MRI = habitat analysis magnetic resonance imaging, ClinicRad = clinical and radiological score.
